# Perioperative Immunosuppressive Factors during Cancer Surgery: An Updated Review

**DOI:** 10.3390/cancers16132304

**Published:** 2024-06-22

**Authors:** Lucillia Bezu, Dilara Akçal Öksüz, Max Bell, Donal Buggy, Oscar Diaz-Cambronero, Mats Enlund, Patrice Forget, Anil Gupta, Markus W. Hollmann, Daniela Ionescu, Iva Kirac, Daqing Ma, Zhirajr Mokini, Tobias Piegeler, Giuseppe Pranzitelli, Laura Smith, The EuroPeriscope Group

**Affiliations:** 1 EuroPeriscope, ESA-IC Onco-Anaesthesiology Research Group, B-1000 Brussels, Belgiumoscardiazcambronero@gmail.com (O.D.-C.);; 2 Département d’Anesthésie, Chirurgie et Interventionnel, Gustave Roussy, 94805 Villejuif, France; 3 U1138 Metabolism, Cancer and Immunity, Gustave Roussy, 94805 Villejuif, France; 4 Department of Anesthesiology, Perioperative and Pain Medicine, School of Medicine, Stanford University, Stanford, CA 94305, USA; 5 Clinic for Anesthesiology, Intensive Care, Emergency Medicine, Pain Therapy and Palliative Medicine, Marienhaus Klinikum Hetzelstift, 67434 Neustadt an der Weinstrasse, Germany; 6 ESAIC Mentorship Program, BE-1000 Brussels, Belgium; 7 Department of Perioperative Medicine and Intensive Care (PMI), Karolinska University Hospital, Solna, 17176 Stockholm, Sweden; 8 Department of Physiology and Pharmacology, Karolinska Institute, 17176 Stockholm, Sweden; 9 Division of Anaesthesiology, Mater Misericordiae University Hospital, D07 WKW8 Dublin, Ireland; 10 School of Medicine, University College, D04 V1W8 Dublin, Ireland; 11 Department of Anesthesiology, Hospital Universitario y Politécnico la Fe, 46026 Valencia, Spain; 12 Perioperative Medicine Research, Health Research Institute Hospital la Fe, 46026 Valencia, Spain; 13 Faculty of Medicine, Department of Surgery, University of Valencia, 46010 Valencia, Spain; 14 Center for Clinical Research, Uppsala University, SE-72189 Västerås, Sweden; 15 Department of Anesthesia & Intensive Care, Västmanland Hospital, SE-72189 Västerås, Sweden; 16 Aberdeen Centre for Arthritis and Musculoskeletal Health (Epidemiology Group), Institute of Applied Health Sciences, Epidemiology Group, School of Medicine, Medical Sciences and Nutrition, University of Aberdeen, Aberdeen AB25 2ZN, UK; 17 Department of Anaesthesia, NHS Grampian, University of Aberdeen, Aberdeen AB25 2ZN, UK; 18 Pain and Opioids after Surgery (PANDOS) ESAIC Research Group, European Society of Anaesthesiology and Intensive Care, 1000 Brussels, Belgium; 19 IMAGINE UR UM 103, Anesthesia Critical Care, Emergency and Pain Medicine Division, Nîmes University Hospital, Montpellier University, 30900 Nîmes, France; 20 Department of Anesthesiology, Amsterdam UMC, 1100 DD Amsterdam, The Netherlands; 21 Department of Anesthesia and Intensive Care, University of Medicine and Pharmacy “Iuliu Hatieganu”, 400012 Cluj-Napoca, Romania; 22 Outcome Research Consortium, Cleveland, OH 44195, USA; 23 Genetic Counselling Unit, University Hospital for Tumors, Sestre Milosrdnice University Hospital Centre, 10000 Zagreb, Croatia; 24 Division of Anaesthetics, Pain Medicine and Intensive Care, Department of Surgery and Cancer, Faculty of Medicine, Imperial College London, London SW10 9NH, UK; 25 Department of Anesthesiology, Perioperative and Systems Medicine Laboratory, The Children’s Hospital, Zhejiang University School of Medicine, National Clinical Research Center for Child Health, Hangzhou 310052, China; 26 Clinique du Pays de Seine, 77590 Bois le Roi, France; 27 Department of Anesthesiology and Intensive Care, University of Leipzig Medical Center, 04275 Leipzig, Germany; 28 Department of Anesthesiology and Intensive Care, San Timoteo Hospital, 86039 Termoli, Italy; 29 School of Medicine, Medical Sciences and Nutrition, University of Aberdeen, Aberdeen AB25 2ZN, UK

**Keywords:** anesthesia, cancer, immune factors, surgery

## Abstract

**Simple Summary:**

Removal of the primary tumor is the best treatment for solid tumor disease. However, perioperative factors such as surgical stress, pain, or anemia disrupt the innate and adaptive antitumor immune response and compromise the postoperative benefits of oncological surgery. Alternative strategies could be implemented during the perioperative period to improve immunosurveillance and reduce the incidence of recurrence. Here, we provide a comprehensive list of immunosuppressive factors occurring during the perioperative period and discuss pharmacological and non-pharmacological methods supporting the immune system, with the aim of assisting clinicians in their practice.

**Abstract:**

Surgical excision of the primary tumor represents the most frequent and curative procedure for solid malignancies. Compelling evidence suggests that, despite its beneficial effects, surgery may impair immunosurveillance by triggering an immunosuppressive inflammatory stress response and favor recurrence by stimulating minimal residual disease. In addition, many factors interfere with the immune effectors before and after cancer procedures, such as malnutrition, anemia, or subsequent transfusion. Thus, the perioperative period plays a key role in determining oncological outcomes and represents a short phase to circumvent anesthetic and surgical deleterious factors by supporting the immune system through the use of synergistic pharmacological and non-pharmacological approaches. In line with this, accumulating studies indicate that anesthetic agents could drive both protumor or antitumor signaling pathways during or after cancer surgery. While preclinical investigations focusing on anesthetics’ impact on the behavior of cancer cells are quite convincing, limited clinical trials studying the consequences on survival and recurrences remain inconclusive. Herein, we highlight the main factors occurring during the perioperative period of cancer surgery and their potential impact on immunomodulation and cancer progression. We also discuss patient management prior to and during surgery, taking into consideration the latest advances in the literature.

## 1. Introduction

Surgical removal of the primary tumor remains the best and most frequent treatment for solid malignancies in combination with chemo-radiotherapy and targeted therapies. Despite curative intention, surgical manipulation by the surgeon, as well as an inevitable injury to vascular and lymphatic vessels, are associated with tumor-cell spread to distant organs, leading to minimal residual disease, recurrence, and metastases. In addition, due to mutations and tumor properties, malignant cells escape the host’s immunosurveillance [1]. The perioperative period is also a specific time during which the immune system is weakened by various factors such as anticancer treatments, anemia, malnutrition, transfusion, inflammatory pain, and physical, psychological, and surgical stresses that impair the physiological innate and adaptive anticancer immune response. Thus, the therapeutic challenge in the perioperative management of surgical oncological patients is to maintain immunocompetence. Much preclinical and clinical evidence suggests that the management of the aforementioned factors, as well as the choice of anesthetic agents, could impact cancer outcomes, especially if these factors are integrated into an enhanced recovery after surgery (ERAS) program [2]. Efforts are now focused on a holistic strategy, including pharmacological and non-pharmacological interventions targeted to improve the patient’s immunomodulation of the surgical stress response and to minimize immunosuppression during the perioperative period in order to avoid cancer progression [3].

In this updated review, we discuss the main perioperative immunomodulatory factors occurring during oncological procedures to highlight the complex interplay between surgery, anesthetic interventions, the inflammatory response, and immunosuppression with the aim of guiding clinicians to design specific strategies adapted to cancer disease and individual patient characteristics.

## 2. Preoperative Factors and Oncological Outcomes

In this first part, we focus on the main preoperative factors likely to modulate the anticancer immune response, with particular attention to factors that can be managed by anesthesiologists prior to surgery.

### 2.1. Modifiable Factors

#### 2.1.1. Value of Prehabilitation Programs in Oncological Care

Prehabilitation is a set of interventions before and between treatment plans to improve overall patient status through exercise, nutrition, and psychological intervention. Most published studies evaluated the effects of optimized nutrition and physical exercise in cancer patients. Nutritional therapy and supplementation may improve the nutritional status of patients in whom cancer disease induces hypercatabolism [4]. Immuno-nutrition is a pharmaco-nutriment associating arginine (to increase the synthesis of lymphocytes), polysaturated fatty acids (to decrease inflammatory response and surgical stress), nucleotides (to restore immune functions), and guar fibers (to stimulate the maturation of lymphocytes and macrophages) [5]. Immuno-nutrition is given for 3 days prior to major oncological visceral surgery. The main benefits are the decrease in postoperative complications and shorter length of hospital stay. However, no study has demonstrated the impact of immuno-nutrition on cancer outcomes [6]. The nature of physical exercises involves repetitive training sessions that challenge whole-body homeostasis with a gradual adaptation of cellular, tissue, and organic systems [7]. While data on biomechanical metabolic and physiologic adaptation in skeletal muscle, heart, and adipose tissue are widely available, information on its effects on tumors is limited [8]. The evidence to date shows that physical exercise benefits the immune system [9]. High levels of infiltrating natural killer (NK) cells, as well as cytotoxic T cells in tumors, are associated with a better prognosis in cancer patients. An eventual benefit of exercise on NK cells might be clinically relevant in the context of cancer, given the important role of this lymphocyte subpopulation in antitumor immunity [10,11]. In addition to the fact that aerobic exercise has a positive effect on slowing tumor growth, it also redirects cytotoxic immune cells to the tumor. This mechanism largely depends on the immune profile of the tumor. Physical exercise also increases the level of chemokines, which attract immune cells and activate NK cells, receptor ligands, and immune checkpoint blockade ligands [12]. Type 1 interferon (IFN) signaling might play a role as well; it has the potential to trigger a whole range of immunological and chemical pathways and enhance the immune response. The cellular senescence combined with physical activity improves tumor immunogenicity, type 1 interferon signaling, and increased infiltration of cytotoxic T cells and NK cells [13]. A few studies examining the influence of exercise on these pathways suggest that exercise can reduce some immunosuppressive factors, such as cellular aging, sarcopenia, and psychological stress in cancer patients [14,15,16]. Metabolic by-products also affect tumor immunity under the influence of exercise. Due to elevated levels of the by-products of aerobic glycolysis, which occurs following cellular metabolism in tumors, lactate accumulates in the tumor microenvironment (TME). Such elevated lactate levels can inhibit the activity of the immune system and prevent the action of cytotoxic T cells. This suppression can be alleviated by exercise, which lowers intratumoral lactate levels [17].

Increasing evidence suggests that exercise triggers immune infiltration and partially alleviates immunosuppressive metabolites as well as enhances immunogenicity [18]. While prehabilitation exercise protocols are hard to track because of their diversity and applicability in different patient populations, they might be an important complementary intervention in cancer care. Further studies are needed in this particular group of patients.

#### 2.1.2. Hyperglycemia and Insulin Resistance

Surgical stress response (SSR) increases plasma levels of cortisol and glucose. In turn, hyperglycemia enhances plasma levels of insulin. Insulin resistance (accompanied by other diabetes-related conditions, such as obesity, adipocytokines secreted by adipocytes and macrophages in adipose tissue, interleukin (IL)-6, tumor necrosis factor (TNF)-α, leptin, retinol binding protein-4, lipocalin-2, and adiponectin that may aggravate insulin resistance) plays a detrimental role in the biology of a tumor and its progression [19,20,21]. Hence, patients with diabetes have significantly increased mortality and postoperative complications as compared with non-diabetic patients [22,23]. It is thus of importance to manage hyperglycemia during the perioperative period of cancer surgery [22]. Interestingly, the antidiabetic drug metformin has demonstrated potent anticancer effects by numerous mechanisms, including directly affecting cancer cells through AMP kinase activation, indirect effects by reducing glucose and insulin levels, inhibiting neutrophil-extracellular-trap-induced oncogenesis, and modifying the TME [24,25]. This is why metformin has been proposed for therapeutic antineoplastic repurposing [25].

#### 2.1.3. Anemia and Allogeneic Blood Transfusion

Cancer patients regularly suffer from tumor- or therapy-associated anemia [26], or perioperative blood loss [27], which might also be associated with a worsened outcome in certain cancer entities [28,29]. These circumstances lead to an increase in blood transfusions in this particular cohort of patients [30]. Although allogeneic blood transfusions might be considered potentially life-saving interventions [31], they also carry certain risks, such as iron overload, hemolytic or non-hemolytic transfusion reactions, anaphylactic reactions, or transfusion-associated lung injury [32]. Additionally, certain evidence also points towards an association between allogeneic blood transfusions and a worsened outcome after surgery, especially in cancer patients [33,34]. The administration of allogeneic blood products might cause a so-called “transfusion-related immunomodulation” (TRIM) [35], which not only leads to the induction of T suppressor and apoptosis in certain immune competent cells but also diminishes the activity of NK cells [35], which play an important role in the detection and elimination of circulating tumor cells (CTCs). As CTCs are released from the tumor during surgical resection [36], a decrease in NK cell activity might enhance CTCs in their ability to escape immunosurveillance and therefore favor the formation of metastases [36,37]. Thus, in a retrospective evaluation of 291 patients undergoing resection of esophageal cancer, Ling and colleagues observed significantly decreased survival in patients who received more than one packed red blood cell (PRBC) unit in the perioperative period [38]. Similarly, a recent Cochrane analysis also demonstrated an association between perioperative blood transfusion and higher recurrence rates after the resection of primary tumors in patients with colorectal cancer [34]. A recent retrospective study evaluating data from pediatric patients undergoing surgery for nephroblastoma was able to show an association between intraoperative PRBC transfusion and a reduction in recurrence-free survival after surgery [39].

The amount of blood loss might be affected by the type of tumor, its size, the stage of the disease, the duration of surgery, or any concomitant comorbidities of the patient [29,40,41]. The ideal regimen to accomplish the goal of a reduction in allogeneic blood transfusions will therefore possibly be the implementation of institutional patient blood management programs [42,43], as these bundles of actions not only include a restrictive blood transfusion approach but also comprehensive management of preoperative anemia and strategies to minimize intra- and perioperative blood loss (e.g., tranexamic acid, iron supplementation) [44,45,46]. It is worth noting that intraoperative use of Cell Saver^®^ represents a risk of tumor cell spread [34,47]. This device can exceptionally be employed with specific leukocyte-reduction filters in case of hemorrhagic shock or during sarcoma removal (without capsular rupture) [48]. Erythropoietin can be indicated in cases of refractory anemia despite appropriate treatment prior to essential chemotherapy [49].

Finally, although blood transfusions might be inevitable in certain situations, the data coming from studies evaluating the association of perioperative blood transfusion and outcome in oncologic patients clearly suggest that a reduction in the utilization of allogeneic blood products might improve survival after tumor surgery [50,51,52,53].

#### 2.1.4. Psychological Stress

The impact of psychoneuroimmunology on the incidence and progression of cancer is a hotly debated topic supported by the emergence of concrete evidence [54]. Psychological stress and depression alter immunity by diminishing the functions of T and NK cells via the activation of the corticotropic axis and the release of catecholamines and glucocorticoids [55]. Many tumor cells express alpha- and beta-adrenoceptors on the plasma membrane [56]. In vitro, the culture of cancer cells in the presence of epinephrine or norepinephrine stimulates proliferation, migration, and survival [57,58]. Interestingly, co-treatment with beta-blockers inhibits the catecholamine-induced tumor effects [59,60].

In an in vivo stress model, mice bearing ovarian tumors were enclosed for 2–6 h in a tube. Plasma levels of norepinephrine and corticosterone, as well as beta-adrenergic activity, matrix metalloproteinase (MMP) 2, MMP9, and vascular endothelial growth factor (VEGF), were significantly elevated in the group of stressed mice compared with the control group. Surprisingly, mice subjected to the same stressed conditions and treated with propranolol showed an abolition of angiogenesis [61]. In a similar model of claustrophobia, stress-stimulated epinephrine production promoted mammary tumor growth in rodents [62].

In a cohort of 65 ovarian cancer patients, the presence of psychological support was correlated with better cytolytic activity of NK cells [63]. The clinical trial by Cui et al. reported that low plasma level of epinephrine was significantly correlated with better recurrence-free and overall survival in breast cancer patients [62]. In line with this, data extracted from retrospective studies in which some patients incidentally received beta-blockers revealed better outcomes after cancer surgery [64]. A few prospective studies also supported that beta-blockers reduced the incidence of recurrence and the risk of progressive disease [65,66]. By blocking stress hormones and thanks to their anxiolytic property, beta-blockers offer the opportunity to alleviate both glucocorticoid and psychological stress.

These preliminary data confirm the relevant role of psychological stress in modulating various aspects of the antitumor immune response and the importance of psychological support and premedication prior to cancer surgery.

### 2.2. Non-Modifiable Factors

#### 2.2.1. Neoadjuvant Anticancer Therapies: Generalities

Although surgery remains the main curative treatment for solid tumors, cancer management is complex and includes individualized neoadjuvant immunotherapy, chemotherapy, and/or radiotherapy. Extending this personalized approach, immunotherapy in the critical perioperative period could be a way to overcome perioperative immunosuppression. This strategy is not approved and rarely discussed in the literature but deserves to be evaluated; the perioperative period is a window of opportunity to minimize the impact of the perfect storm of tumor cell circulation and immunosuppression [67]. Classically, a minimum of 3 weeks must be allowed between neoadjuvant therapy and surgery due to the potential impact on wound healing and immune function. But nowadays, there are clear and positive experiences with some strategies and several tumor types, such as hyperthermic intraperitoneal chemotherapy (HIPEC) [68].

#### 2.2.2. Neoadjuvant Chemotherapy

The major concerns are related to the effects of chemotherapeutic agents on wound healing and immune function, but both these are closely related to the type of drug administered. Anti-VEGFA (bevacizumab) and alkylating agents (cisplatin, cyclophosphamide) may increase the risk of anastomotic leakage and infection in addition to the direct impact on the patient’s status [69]. But the effects of antimetabolites (methotrexate and 5-fluorouracil) or antibiotics with antitumor activity (doxorubicin, mitomycin C) are less clear. Despite this theoretically low risk with selected agents and effective perioperative inclusion of some types of chemotherapy as in HIPEC, long periods of immunosuppression have been reported following some types of chemotherapy, and therefore they are not the first line of management today [70].

#### 2.2.3. Neoadjuvant Immunotherapy

The goal of immunotherapy is to enhance cellular and/or humoral immune responses to minimal residual disease by several pathways: immunogenic cell death, antibody-dependent cellular cytotoxicity, and complement-dependent cytotoxicity [71,72]. Like chemotherapy, immunotherapy has been avoided close to surgery, as its side effects can increase postoperative complications, some of them due to an increased risk of infection or sterile inflammation. However, the benefits may outweigh the risks [73]. Perioperative immunotherapy could potentially increase the therapeutic benefit during this time frame, in contrast to a later use during the course of cancer management. The ideal immunotherapy drug must induce rapid activation of the immunological response, lack tumor-promoting effects, have minimal contraindications for surgery, and low stress response. The immunotherapies that best fit these considerations are the toll-like receptor (TLR) 9 agonist CpG-C and the TLR4 agonist glucopyranosyl lipid A. The perioperative use of these immunostimulant agents could probably be well tolerated, but further studies are warranted to identify the best strategy [74].

In summary, preoperative factors specific to cancer disease and patients’ characteristics can weaken the immune system and reduce the expected curative effect of surgery. Some of these factors, such as anemia, malnutrition, hyperglycemia, or lack of physical exercise, can easily be corrected in a prehabilitation program, which will need to be designed by a multidisciplinary team to best suit each patient. Conversely, the immune consequences of neoadjuvant treatments are difficult to avoid, and anesthetists need to be aware of this so as not to worsen outcomes.

## 3. Perioperative Factors and Oncological Outcomes

In this second part, we summarize the surgical factors that might sustain tumor progression. We also discuss the potential immunomodulating properties of anesthetics, which could promote or inhibit tumor cell signaling pathways.

### 3.1. Surgical Stress Response and Oncological Outcomes

Surgical stress response (SSR) is a pattern of physiological and pathophysiological responses to the trauma of surgery, which occurs despite analgesia and anesthesia. Traditionally, it comprises two broad components: neuroendocrine-metabolic response and inflammatory-immune response.

**Figure 1:**
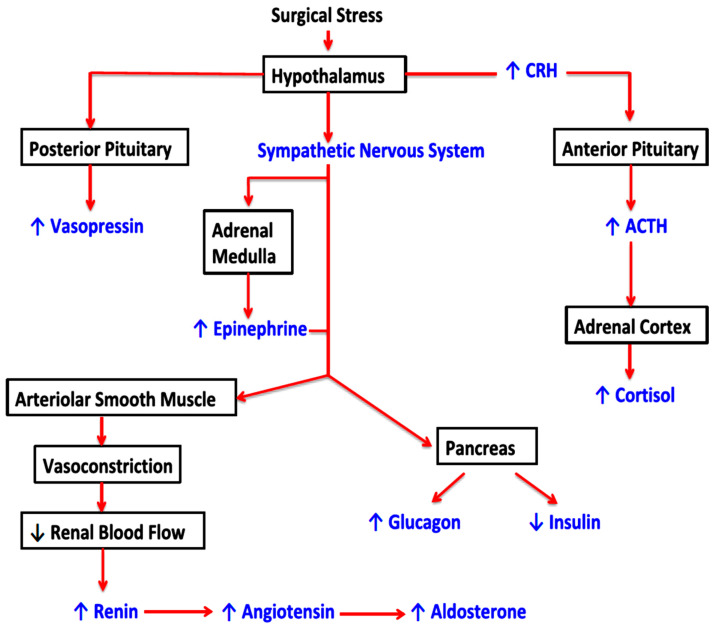
Integration of the stress response to surgery by the hypothalamus, sympathoadrenal system, and sympathorenal system [corticotrophin-releasing hormone (CRH), adrenocorticotropic hormone (ACTH)]. This figure is reproduced with permission from Elsevier; it was published in BJA Education, Volume 20, Issue 9, by Cusack et al., titled ‘Anaesthesia, analgesia, and the surgical stress response’, pages 321–328 [75], Copyright Elsevier (2020).

The extent of the SSR is related to the magnitude, invasiveness, and duration of the surgery, which would include many types of major cancer surgery [75]. Figure 1 summarizes how SSR activates the neuroendocrine-metabolic response via the hypothalamic–pituitary–adrenal (HPA) axis, the sympathetic nervous system, and the renin–angiotensin system, with potentially deleterious effects, including activation of a systemic inflammatory response syndrome, hypercatabolism, hyperglycemia, acute kidney injury, muscle wasting, and impaired wound healing. Recent work has also introduced an evidence-based nuance to this traditional description of SSR. Preoperative co-morbid patient factors, such as fasting, cardiovascular deconditioning, psychological stress, and underlying cancer pathology itself, accompanied by frailty (in up to 70% of patients) or cardiovascular deconditioning (from chemotherapy for cancer) [76], may cause chronic HPA dysfunction before the SSR, characterized by high preoperative adrenal cortisol production independently of the HPA [76]. The frequent development of postoperative complications also adds to the complexity: starvation, impaired mobility, and sepsis after surgery may also cause HPA dysfunction. In patients with a dysfunctional HPA, perioperative cortisone supplementation may improve postoperative outcomes [77]. These premises suggest that physical exercise in the context of prehabilitation may alleviate some of the deleterious effects of SSR.

The inflammatory trauma of surgery is also responsible for the release of damage-associated molecular patterns (DAMPs) that include cytokines, high-mobility group box 1 protein (HMGB1), heat shock proteins, hyaluronan fragments, ATP, uric acid, and heparin sulfate in the operative site. These DAMPs activate TLRs, which in turn increase the regulatory transcription factor NF-κB via intracellular signaling and then evoke an innate immune response and release of pro-inflammatory cytokines [78]. HMGB1 is a nuclear protein that is involved in pathophysiology, signaling the production of pro-inflammatory cytokines and chemokines through TLRs and advanced glycation end-products. Being present both intracellularly and extracellularly, HMGB1 triggers inflammation through direct binding to TLR4 or by complex formation with exogenous or endogenous molecules in the cytoplasm or extracellular space [79]. Major surgical trauma induces cell injury and death, which subsequently results in extracellular histone release, causing direct cytotoxicity to epithelial and endothelial tissue and also the mediation of TLR9 activation, which induces significant pro-inflammatory cytokine (TNF-α and IL-6) release [80]. Cell injury and death release ATP into the extracellular space, where it has the ability to initiate inflammation [81]. All these DAMPs initiate systemic inflammation, which consequently stimulates the local spread of cancer cells at the surgical site and CTCs to further develop into metastasis [82]. Tumor-associated macrophages (TAMs) and neutrophils (TANs) and their associated derivatives, especially in the TME, are integrated into cancer-related inflammation to promote tumor progression. Meanwhile, tumor cells release cyto-toxicants that can directly damage the normal tissue and release DAMPs, further promoting cancer progression [83]. Indeed, the TME is a complex ecosystem, where a delicate balance exists between tumor-promoting and anti-tumoral immune components. Inflammation can modulate and increase the pro-tumor/anti-tumor quotient, resulting in tumor progression and invasion.

**Figure 2:**
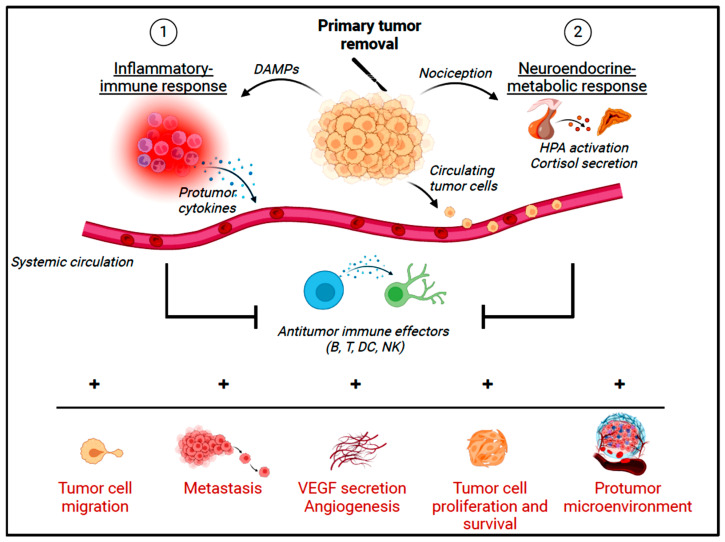
How the stress response to surgery during tumor resection might inadvertently promote transient immune impairment, cancer cell survival, and subsequent metastasis. Abbreviations: B, B lymphocyte; DAMPs, danger-associated molecular patterns; DC, dendritic cell; HPA, hypothalamic–pituitary–adrenal axis; NK, natural killer; T, T lymphocyte; VEGF, vascular endothelial growth factor. Created with www.BioRender.com.

In response, surgical inflammation affects both the innate and acquired immune systems. The innate immune response involves phagocytic cells, such as neutrophils, macrophages, and dendritic cells, producing pro-inflammatory cytokines (IL-1, IL-6, and TNF-ɑ). These cytokines, especially IL-6, are responsible for systemic changes included in the inflammatory response [84] and, depending on the pattern, for postoperative complications and disease-free and overall survival [85]. Several studies have shown that anesthetic agents may influence IL-6 levels [86,87]. Cytokine patterns initiated by the innate immune response determine what type of adaptive immune response is initiated. The so-called T helper 2 (Th2) response is effective in eliminating bacteria. In contrast, a T helper 1 (Th1) response is dominated by cytotoxic cells that detect and kill tumor cells. However, the SSR seems to enhance the Th2 response at the expense of a weakened Th1 response, potentially facilitating residual cancer cell survival despite surgery [75,88] (Figure 2).

This initial inflammatory-immune response is followed by a compensatory period of immunosuppression [89], mediated by cortisol, anti-inflammatory cytokines (IL-10), and a shift towards a Th2 > Th1 response. Depression of T- and NK-cell cytotoxicity is pronounced and can last for several weeks, further increasing the patients’ susceptibility to potential cancer progression through tumor cell immune evasion [90].

Thus, suppressing inflammation perioperatively could prove to be beneficial. A few clinical trials identified the beneficial effects of non-steroidal anti-inflammatory drugs (NSAIDs) during cancer surgery. Four retrospective studies indicated that dexamethasone and NSAIDs alone or in combination used just before or during surgery can improve long-term outcomes, including survival in patients with lung or breast cancer following surgery [91,92,93,94]. The mechanisms through which NSAIDs might improve oncological outcomes have been partially deciphered. Their anti-cyclooxygenase (COX)2 property could participate in decreasing surgical stress and the tumor’s inflammatory environment. Furthermore, some preclinical studies have demonstrated that ketorolac, an anti-COX1, and COX2, impaired the angiogenesis and proliferation of malignant cells by inhibiting the oncogene *c-Myc* and modulating the signaling pathways mediated by TSP-1 and HIF-1α [95,96]. Ketorolac also slowed down tumor growth in cancer models established in mice [95].

However, surgical-trauma-related systemic inflammation is a double-edged sword: one side is important for wound healing, and on the other, it is a risk factor causing cancer recurrence and metastasis. How to balance this response for better status in cancer patients is an important question to be answered in clinical practice. Of note, in case of major surgical complications, SSR associated with bleeding, ischemia, and reperfusion injury (IRI) may lead to a systemic inflammatory response syndrome (SIRS) with the risk of multiple organ failures [97].

### 3.2. Could Anesthetics Alleviate Perioperative Immunosuppression and Prevent Recurrences?

#### 3.2.1. Opioids

For decades, it has been known that opioids affect various aspects of anticancer immunity, in particular lymphocyte proliferation and function, including effects on cytokine production and cytotoxicity [98,99]. Opioid receptors are also expressed on the membrane of tumor cells, affecting their metabolism [100]. Interestingly, all these characteristics are related to effects that are dose- and opioid type-dependent. As a result, these effects may be modified during the perioperative period by using opioid-sparing strategies or opioid-free techniques [101]. Given the complexity of the picture, research has emerged on links between oncological outcomes and immunological biomarkers in cancer surgery (Table 1).

A recent review on opioids used in cancer surgery found eight randomized controlled trials (RCTs) and five ongoing clinical trials. Immunological parameters, primarily the neutrophil-to-lymphocyte ratio, but also cytokines and other mediators, were found to be modified (Table 2).

The review also identified ongoing trials evaluating markers more directly related to tumor cell activity: caspase-3 (a marker of apoptosis), hypoxia-inducible factor (HIF)-1α, NF-κB (transcription factors associated with cell proliferation), and VEGF. Conflicting results were found regarding effects on cancer cells, with significant heterogeneity among studies [107,108]. Opioids favored apoptosis by increasing the expression of *p53*, a tumor suppressor gene, and its associated proteins [109], in particular protein kinase B [110] and mammalian target of rapamycin kinases [111]. Some preclinical studies observed that morphine did not facilitate disease progression or invasion [112]. Conversely, some evidence suggests that opioids could encourage carcinogenesis by promoting the activity of MMP enzymes by increasing the expression of the oncogene *c-Myc* while decreasing the anti-angiogenic gene *TSP-1* [96,113]. No conclusive evidence was found on clinical oncological outcomes [114].

All in all, anesthetic modalities, with or without opioids, may differently affect anticancer immune-related biomarkers. However, no good evidence has been generated on the links between these effects and long-term outcomes. Future trials will have to improve aspects of study methodologies, including conditions (i.e., cancer subtypes), standardization of definitions, and documentation of outcomes (like cancer recurrence). These essential endpoints are often defined differently, and other important outcomes, like quality of life, are frequently not documented.

#### 3.2.2. Local Anesthetics and Tumor Biology

Local anesthetics (LAs) are traditionally seen as sodium channel blockers that prevent nerve conduction, leading to anesthesia [115]. However, LAs also have strong anti-inflammatory effects [116,117,118] and reduce signaling related to the SSR [119,120], while immunomodulation by LAs might have the potential to influence tumor biology [36,121,122]. Several mechanisms have been suggested underlying the potential tumor-protective effects of LAs. First, LAs might inhibit immune suppression induced by surgery, e.g., by preserving NK cell function [123]. Some reports suggest that LAs might reduce the activation of neutrophils or macrophages [124]. Surprisingly, CTCs are able to exploit the functions of both these immune cells to extravasate. Thus, LA-mediated immune suppression might be beneficial to halt the migration of CTCs [125]. Second, currently used amide-linked LAs have been shown to preserve endothelial barrier function by attenuation of TNF-α-induced activation of Src tyrosine protein kinase [126,127,128], thus limiting CTCs from extravasating and forming metastases in remote locations [118]. Third, inhibition of TNF-α signaling might reduce the invasiveness of tumor cells [129], attenuate the ability of neutrophils to bind to tumors as well as to endothelial cells [118], and limit the extravasation of CTCs [125]. Most of these effects seem Na^+^-channel independent, although for some cancer types, e.g., in colon cancer [130], a Na^+^-channel-mediated effect cannot be ruled out. Fourth, LAs might also affect tumor cell viability and proliferation. Treatment of human breast [131], colorectal [132], ovarian [133], and lung cancer [134] cells with LAs has also revealed a significant inhibition of survival and proliferation via multiple pathways, which have not been fully elucidated. Recent evidence also points towards a possible synergistic effect of LA with chemo- [135] or immuno-therapy [136].

These promising and mostly experimental findings have not been translated into improved outcomes in clinical studies when LA has been used during regional anesthesia techniques [137,138]. The discrepancy between laboratory conditions and the much more complex “real-life” tumor biology, including the TME, might explain the current mismatch [139]. First, evidence that LAs might influence the TME has been provided by a recent study assessing the effects of a preoperative peritumoral infiltration with lidocaine vs. no intervention in breast cancer patients, whereby a significantly prolonged survival rate was observed in the lidocaine group [140]. However, as there were several limitations to this particular study, including the lack of a placebo control, the results should be interpreted with caution [140].

The route of administration of the LA might also be important. Intravenous administration of LA was shown to have anti-inflammatory effects [119] and might be beneficial in patients with cancer [122]. LA might also be administered via other routes that lead to an effective LA concentration at the tumor site. In a recent small, randomized, double-blind pilot study, intraperitoneal lidocaine or placebo was administered during 48 h following cytoreductive surgery for ovarian cancer. Adjuvant chemotherapy, a surrogate for better long-term outcomes, could be started significantly earlier in the group receiving LA [141]. However, the use of LA intravenously for this indication must be considered off-label and is, unfortunately, still not recommended in many countries due to concerns regarding LA toxicity. It is worth noting that the concentration of lidocaine that was used in all clinical trials mentioned before was associated with a very low risk of toxicity, even in small children and infants [142].

In conclusion, the question of whether LAs might clinically influence tumor biology in a meaningful way has not yet been answered. When used in the context of regional anesthesia, their beneficial effects seem to be limited. Future research should focus more on the interaction of LAs with the tumor itself and its microenvironment, as well as on the underlying mechanisms. In clinical trials, intravenous or peritumoral (local infiltration or intraperitoneal administration) should be further explored.

#### 3.2.3. Inhalational Anesthesia

It is widely acknowledged that volatile anesthetics possess pro-inflammatory and immune-modulating properties, which could potentially favor cancer recurrence [143]. Volatiles might impair the immune system and facilitate metastatic dissemination of residual cancer cells. Yet, the exact molecular mechanisms underlying these effects remain incompletely comprehended.

Volatiles have been shown to affect both innate immunity components, such as peripheral blood mononuclear cells, neutrophils, NK, monocytes, dendritic cells, and tissue-resident macrophages; adaptive immunity by reducing lymphocyte proliferation and by increasing lymphocyte apoptosis; and also humoral immunity [144,145]. Volatiles can directly affect receptors located on the surface of cells involved in the immune response, including calcium/magnesium ion channel proteins, integrin β2, Ras1 protein, and TLR. This interaction can trigger degranulation of cells in the immune response, such as macrophages and NK cells, leading to a reduction in their cytotoxic abilities and inhibition of anti-tumor effects. One of the immune cell populations most affected by perioperative medications is NK cells. NK cells act as primary effectors against cancer. Unlike cytotoxic CD8^+^ T cells, NK cells respond rapidly to targets with heightened cytotoxicity, yet they have lower immunogenicity and may not need additional ligand-driven activation. Hence, there is growing recognition of the potential significance of NK-cell-based therapy in the development of future immunotherapies [146]. Volatiles inhibit the cytotoxicity of NK cells and can trigger apoptosis in T-lymphocytes that drive immune surveillance and anti-metastatic immunity following cancer surgery. Sevoflurane enhances the generation of intracellular reactive oxygen species (ROS) and induces apoptosis in lymphocytes [147]. Through dose-dependent mechanisms, desflurane and sevoflurane prompt apoptosis within the T cells located in the thymus. Additionally, desflurane enhances apoptosis in B lymphocytes by activating inositol triphosphate, potentially contributing to immune suppression following surgical procedures [148]. Both isoflurane and sevoflurane can reduce mitochondrial membrane potential in a dose-dependent fashion, resulting in cytochrome C liberation and apoptosis by caspase-3 [149]. It is plausible to assume that mitochondria are the primary effectors of volatile anesthetic-induced apoptosis and decline in immune cell count induced by isoflurane [148]. Remarkably, propofol could potentially mitigate the mitochondria-related apoptosis triggered by sevoflurane [147]. The lymphocyte function-associated antigen-1 integrin is fundamentally responsible for the trafficking and infiltration of immune cells and is bound by sevoflurane and isoflurane. They disrupt its interaction with its primary binding companion, intercellular adhesion molecule-1, on antigen-presenting cells. This interference leads to the inhibition of immune cell adhesion [150]. In addition, while sevoflurane upregulates HIF-1α activity, halothane and isoflurane downregulate it [151].

Mice exposed to sevoflurane exhibit elevated levels of proinflammatory cytokines triggered by the activation of the IL-6/JAK/STAT3 pathway that is associated with tumor metastasis [152]. A human clinical trial demonstrated that sevoflurane induces apoptosis-inducing factor levels in cardiac surgery patients, correlating with decreased lymphocyte counts [153]. However, volatiles can enhance immunity in acute kidney injury models when the dosage and exposure duration are optimized [144].

To date, research has not established a definitive link between volatiles, immune suppression, inflammation, and cancer recurrence. At a clinical level, basic research results have to deal with the contradictive results of retrospective studies and RCTs [144]. It seems, however, that volatiles might have a more negative impact on the overall survival of cancer patients compared to intravenous anesthetics. In a retrospective analysis of 7030 patients, the 3-year survival was increased with a hazard ratio of 1:80 with inhalational anesthesia compared to intravenous anesthesia [154]. This may suggest that propofol might be a preferable choice for anesthesia during tumor excision surgeries. There is a need for further basic research investigating the molecular mechanisms involved in volatile anesthetic action during the perioperative period of cancer surgery and also for prospective RCTs evaluating immunity and inflammation and cancer recurrence after anesthesia using inhalational anesthetics [144].

#### 3.2.4. Intravenous Anesthetic Agents

Propofol can exert a direct antitumoral effect on various cancer types [155]. Its action includes inhibition of cancer angiogenesis, invasion, dissemination, proliferation, and cancer cell apoptosis. Propofol can affect both epigenetic pathways (miRNA, methylation, lncRNA, acetylation) and inhibit the release of extracellular vesicles during cancer resection; it also affects signaling pathways such as HIF-1α, NF-κB, amphiregulin, a disintegrin and metalloproteinases family (ADAMs), mitogen-activated protein kinases (MAPKs), SNAIL-related zinc-finger transcription factor (SLUG), and nuclear factor erythroid 2-related factor 2 (Nrf2). By blocking MAPK, propofol transiently hinders the function of HIF-1α. Angiogenesis is activated by HIF-1α, which correlates with heightened cancer aggressiveness and worsened clinical outcomes [156].

On the other hand, propofol demonstrates anti-inflammatory and antioxidative properties [157]. Propofol acts as an anti-inflammatory agent by downregulating pro-inflammatory cytokines, cyclooxygenases (COX), prostaglandin E2 (PGE2), and IFN-γ [158]. Propofol reduces serum tumor angiogenesis-related factors, such as VEGF-A and C, and tumor growth factor (TGF)-β in non-small-cell lung cancer patients following surgery [159]. It also has a protective action against postoperative immunosuppression, as it has no impact on IL-2/IL-4, Th1/Th2, or CD4^+^/CD8^+^ T cell ratios [158]. Moreover, propofol increases cytotoxic T-lymphocyte activity and the Th1/Th2 balance towards Th1-like response [160]. The drug also demonstrates various effects on many immune cell types: propofol can inhibit COX2 activity and the production of PGE2, an immunity suppressor, from monocytes and dendritic cells, but also promote immune function by increasing TNF-α and IL-1 [155]. Propofol can suppress M1 macrophage activity but not M2 activity, and consequently prevent inflammation. The action of propofol on neutrophil and macrophage chemotaxis, phagocytosis, and ROS is dose-dependent. Propofol may enhance postoperative NK cytotoxicity in esophageal squamous cell carcinoma patients. It does so by increasing IFN-γ secretion by NKs, which inhibits PGE2 production by macrophages. This cascade can lead to an increased synthesis of IL-12 and IL-18, further activating NKs and enhancing their cytotoxicity against tumors. These findings are consistent with previous research demonstrating propofol’s ability to induce IFN-γ. Observational studies have shown a potential decrease in cancer recurrence and better survival with propofol compared to inhalational anesthetics [154,161]. Compared to sevoflurane and opiates, propofol and paravertebral anesthesia reduce MMP and pro-tumorigenic cytokines after breast cancer surgery [162]. Propofol, compared to volatiles, produced less immunosuppression in patients receiving laparoscopic radical hysterectomy following cervical cancer [163]. While conflicting findings exist, they stem mainly from small, low-quality studies. Several trials comparing inhalational anesthesia to intravenous propofol anesthesia are ongoing and will provide clearer evidence of their action in cancer recurrence after surgery [164].

The benefits of alpha-agonists such as clonidine and dexmedetomidine remain debated in surgery. Dexmedetomidine could exert both protumor or antitumor effects in vitro and in vivo through various mechanisms involving apoptotic and angiogenic signaling pathways as well as epigenetic changes [165]. Recent insights revealed that dexmedetomidine could decrease pro-inflammatory markers such as IL-6 and TNF-α, alleviate corticoid stress, and support the immune system by enhancing the CD8^+^/CD4^+^ ratio and favoring the synthesis of the cytolytic IFN-γ in clinical trials [165]. Dexmedetomidine could thus offer the dual advantage of being antitumoral and opioid-sparing.

#### 3.2.5. Choice of Anesthetics for Onco-Surgery: A Future Challenge?

The hypothesis that long-term survival after cancer surgery will be affected by the choice of anesthetics seems bizarre. Leaving small studies aside, two retrospective studies, the first on breast and colorectal cancer (*n* = 2838) [161] and the second on a mix of different cancers (*n* = 7030) [154], revealed that in both studies, breast cancer was not affected by the choice of anesthetics, while it appeared to have surprisingly large effects on survival in other cancers, favoring propofol. The difference in overall survival was, e.g., 7.2 percentage points (p.p.) after propensity score matching (*n* = 5214) in the second study. This would correspond to the effect of adding not just one but several cytotoxic drugs to the treatment. Many retrospective studies and several meta-analyses [166,167,168,169,170,171] in the absence of properly sized RCTs have been conducted. One of the meta-analyses consisted of 20 small RCTs, including 2021 patients with breast cancer [166]. The two-year recurrence-free survival rate was higher in the propofol group, but the overall survival was not affected significantly. The most recent meta-analysis included 44 studies with 686,923 patients [172]. Only two of these studies were RCTs (*n* = 2955 patients) [173,174]. The conclusion was that “propofol-based anesthesia may have a beneficial effect on overall survival and recurrence-free survival compared to inhalation anesthesia in some cancer operations”. Of the two published RCTs, one included 1670 patients with breast cancer [173]. Here, the sample size was calculated based on data from a retrospective study and on national Swedish survival data. The effect size was based on a minimum difference in overall survival of 5 p.p. The second RCT included 1195 patients with a mixture of different cancers, dominated by gastrointestinal cancers [174]. The effect size was set for the entire patient population, neglecting the differences in survival between different types of cancer. Here, a minimum survival difference of 10 p.p. was assumed. No difference in long-term survival between anesthetics has been found in either of the two RCTs. A risk of type II error due to unrealistically large effect estimates should be considered. A third RCT, not included in the latest meta-analysis, addressed breast cancer [137]. A combination of the assumed most tumor-protective anesthetic strategy, propofol combined with a paravertebral block, was compared to the supposed most tumor-promoting anesthetic strategy, namely the combination of sevoflurane and opioids. Here, the most tumor-preventing anesthetic strategy did not perform better than the more detrimental one with respect to either primary or secondary tumor outcomes. Although solid conclusions based on this study can only be drawn for breast cancer surgery, the results reflect nicely the very low a priori probability that the choice of the anesthetic regimen could clinically relevantly affect survival or tumor-related outcome.

The question still remains whether we have to set out to determine for each hypnotic and all opioids their specific effects according to tumor types or whether contradictory effects in the preclinical setting and solid data from a large RCT in breast cancer are sufficient to follow the a priori probability, that it is rather unlikely that a specific anesthetic regimen might exert clinically meaningful effects on tumor biology [175].

## 4. The Postoperative Period: Specificities

### 4.1. Postoperative Acute Pain

Evidence suggests that acute pain alters the behavior and effectiveness of NK cells. Therefore, surgical procedures may be deeply immunosuppressive when associated with severe pain. In the preclinical study by Sacerdote et al., an electrical stress (1.6 mA) was applied under the paws of rats. The cytotoxic function of NK cells was significantly altered in 30 min and partially restored 2 h after acute pain [176]. In the in vivo study by Tsuchiya et al., the number of lung metastases was directly correlated to the invasiveness of surgery and facilitated by the activity of MMP [177]. The surgical rat model of Page demonstrated that the suppression of NK cell activity enhanced the metastatic spread, while pain control decreased it [178]. In humans, the clinical trial of Fujisawa et al. confirmed the association between better recurrence-free and overall survival and NK activity after lung cancer surgery [179]. In summary, by impeding the activity of NK cells, surgery could encourage refractory residual cancer cells. Whether acute pain control can improve oncological outcomes during oncological procedures remains an open question.

In this case, the choice of analgesics could represent a new challenge for onco-anesthetists. As opioids could be protumor agents, multimodal analgesia combining different molecules and modes of administration (local anesthetics, ketamine, infiltration, anti-inflammatory drugs…) will probably be implemented more widely in the future in the first postoperative days.

### 4.2. Detection and Optimized Management of Immunosuppressive Complications

Similarly to the preoperative period, many immunosuppressive factors can occur after the procedure, such as anemia due to bleeding, malnutrition in the case of artificial nutrition, or malabsorption (after colon or pancreas removal, for instance). Some surgeries lead to expected complications, which may alter the immune effectors (e.g., glycemic variability, metabolic troubles, neutropenia after HIPEC) [180]. Furthermore, the postoperative period is favorable to nosocomial infections due to the impairment of the immune system and the disturbance of the microbiota by chemotherapy, antibiotics, artificial nutrition… Non-specific strategies of rehabilitation have to be quickly implemented, such as physical and respiratory physiotherapy, mobilization, preventive anticoagulation, etc., as well as specific protocols according to the surgery, such as injection of growth factor G-CSF 3 days after HIPEC to avoid neutropenia or nutritional supplementation. It is also essential to continue the withdrawal of alcohol, tobacco, and other addictions, which are very harmful to the immune system, by prescribing benzodiazepines, hyper-hydration, nicotine substitutes, or methadone [181]. The prevention, early detection, and management of immunosuppressive complications are mandatory to minimize the length of stay and improve overall and recurrence-free survival. Indeed, the faster the recovery, the sooner adjuvant treatments can be considered.

### 4.3. Expert Team in Oncological Surgery

Cancer surgery involves a wide range of specificities related to the patient, the disease itself, its pathophysiology, consequences, and treatments. Given the variety of cancer types and the outstanding diversity and specificity of current anticancer treatments, it is essential that the patient is hospitalized during the postoperative period in a department adapted to the intensity of the surgery (intensive care, ward, etc.), and cared for by an expert team trained to recognize complications and immunosuppressive factors after cancer surgery. Postoperative oncological care needs a multidisciplinary team involving not only the surgeon–anesthetist duo but also a physiotherapist, psychologist, stoma-therapist, nutritionist, and oncological nurse. The help of algologists may also be required, specifically in case of acute pain in patients suffering from chronic pain, opiate addiction, or with multiple prescribed analgesics. Because cancer affects all specialties and all ages, and because its incidence is constantly increasing, all anesthetists and healthcare professionals are called upon to care for cancer patients. The introduction of specific courses, teachings, or workshops should become a common and regular practice in hospitals, as well as at conferences and meetings [182].

### 4.4. Impact of the ERAS Program on Oncological Outcomes

In the 1990s, Prof. Henrik Kehlet devised the first ERAS programs corresponding to the simultaneous implementation of the aforementioned perioperative strategies to minimize surgical trauma, reduce complications, and promote fast recovery. Various observational studies and meta-analyses have demonstrated that the ERAS approach is similar whatever the etiology of the disease (benign or malignant), and does not increase readmission or mortality rates in cancer surgery [183,184,185]. Conversely, the oncological ERAS program improves short-term outcomes with a significant reduction in readmission, complications, global morbidity, and immediate mortality. For instance, the optimal control of acute pain and its risk factors implemented in the ERAS program, with the use of paravertebral block during mastectomy and specific management in case of preoperative opioid and alcohol consumption, significantly reduces the use of opioids and the risk of chronic postoperative pain [186]. The awareness of appropriate nutrition and supplementation in cancer patients prior to surgery has also led to a considerable improvement in postoperative outcomes [187]. Furthermore, some clinical studies observed that ERAS could also improve long-term outcomes by enhancing overall survival if 70–90% of the ERAS program was achieved [2]. The underlying mechanisms are various and include, among other things, improvement in perioperative nutrition and a decrease in glucocorticoid stress and inflammatory response thanks to minimally invasive procedures or early resumption of adjuvant treatments [188,189].

## 5. Conclusions

**Figure 3:**
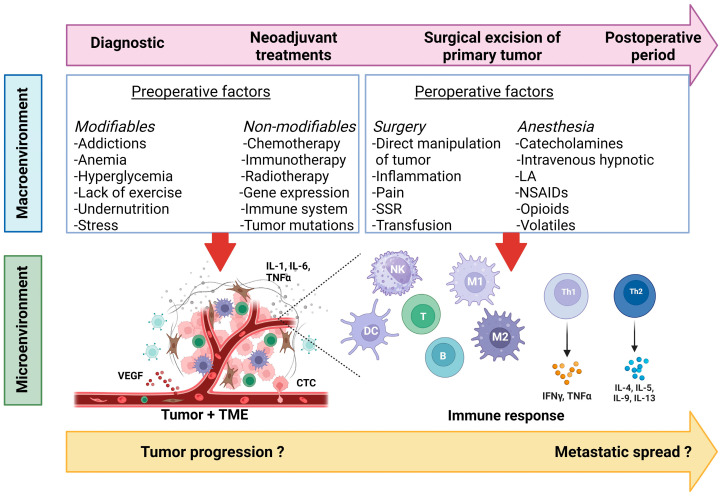
Perioperative immunomodulatory factors during cancer surgery. Abbreviations: CTC, circulating tumor cell; DC, dendritic cell; IFN, interferon; IL, interleukin; LA, local anesthetic; NK, natural killer; NSAIDs, non-steroidal anti-inflammatory drugs; SSR, surgical stress response; TME, tumor microenvironment; TNF, tumor necrosis factor; VEGF, vascular endothelial growth factor. Created with www.BioRender.com.

Several immunomodulatory factors interfere with the antitumor response mediated by the innate and adaptive immune effectors during the perioperative period of cancer surgery (Figure 3). Individually, each factor has a slight impact on the oncological outcome; however, optimal control of all of them could significantly potentiate the immune response. Future research is expected to consider the synergistic effects of these immunomodulatory factors on the antitumor immune response and to design personalized perioperative care incorporating the control of immunosuppressive factors tailored to the characteristics of the cancer disease and the patient’s immunity. Of note, recommending certain anesthetics in onco-anesthesia protocols is still debated due to the methodological difficulty of determining their implication, or lack thereof, in oncological outcomes, and the role of acute pain in tumor progression remains unclear.

## Figures and Tables

**Table 1:** Perioperative immunological parameters studied in cancer surgery. Adapted from Abbas et al. (2018) [102] and Smith et al. (2022) [103].

Immunological Parameter	Description
Neutrophil-to-lymphocyte ratio Platelet-to-lymphocyte ratio Lymphocyte-to-monocyte ratio	Neutrophils: can either promote or inhibit cancer development and growth. Lymphocytes: key components of the adaptive immune system. Platelets: facilitate metastatic spread through production of adhesion proteins, clotting factors, and various interactions. Monocytes: innate immune cells, which can have both pro-tumor and anti-tumor effects.
IL-4	Anti-inflammatory
IL-6	Pro-inflammatory
IL-8	Pro-inflammatory
IL-10	Anti-inflammatory
IL-12	Pro-inflammatory
IL-17A	Pro-inflammatory
TNF-α	Pro-inflammatory
Oxidative stress profile (lipid peroxidation status and antioxidant capacity of plasma)	Lipid peroxidation: oxidative degradation of lipids mediated by free radicals; can cause DNA damage.
CRP	Non-specific marker of inflammation.
NK and CD8^+^ T cells	Cytotoxic function.
T helper cells	CD4^+^ helper cells, enhance function of CD8^+^ cells and macrophages.
Caspase 3	Marker of apoptosis.
Cortisol	Glucocorticoid hormone with immunosuppressive effects.
HIF-1α	Regulates transcription of genes involved in cell viability and proliferation.
VEGF	Growth factor promoting angiogenesis.
NF-κB	Involved in regulation of inflammation, cell activation, and proliferation.

Abbreviations: CRP, C-reactive protein; HIF-1α, hypoxia-inducible factor 1-alpha; IL, interleukin; NF-κB, nuclear factor-kappa B; NK, natural killer; TNF, tumor necrosis factor; VEGF, vascular endothelial growth factor.

**Table 2:** Main findings of published randomized controlled trials comparing opioid-free anesthesia (OFA) to opioid-based anesthesia (OBA). Adapted from Smith et al., 2022 [103].

Outcomes	Immunological		Oncological
Authors	Parameters	Main Findings	Main Findings
Rangel et al. (2021) [104]	Neutrophil-to-lymphocyte ratio	Postoperative neutrophil-to-lymphocyte median rates not significantly different between groups.	Biochemical recurrence-free survival. No significant differences in either outcome between groups.
Aboalsoud et al. (2021) [105]	IL-10, TNF-α, caspase 3	OFA vs. OBA: Increase in IL-10 and caspase 3; decrease in TNF-α.	N/A
Titon et al. (2021) [106]	IL-4, IL-12, IL-17A, TNF-α, oxidative stress profile	OFA vs. OBA: Decrease in lipid peroxidation, increase in antioxidant capacity of plasma. No variation in IL-4, IL-17A, and TNF-α.	N/A

Abbreviations: IL, interleukin; N/A, not applicable; OBA, opioid-based anesthesia; OFA, opioid-free anesthesia; TNF, tumor necrosis factor.

## Data Availability

No new data were created for this review.

## Abbreviations

ADAMs, a disintegrin and metalloproteinases family; COX, cyclo-oxygenase; CRP, C-reactive protein; CTCs, circulating tumor cells; DAMPs, damage-associated molecular patterns; HIF-1α, hypoxia-inducible factor 1-alpha; HIPEC, hyperthermic intraperitoneal chemotherapy; HMGB1, high-mobility group box 1 protein; HPA, hypothalamic–pituitary–adrenal; IFN-γ, interferon γ; IL, interleukin; IRI, ischemia and reperfusion injury; LAs, local anesthetics; MAPKs, mitogen-activated protein kinases; MMP, matrix metalloproteinase; N/A, not applicable; NF-κB, nuclear factor-kappa B; NK, natural killer; NRF2, nuclear factor erythroid 2-related factor 2; NSAIDs, non-steroidal anti-inflammatory drugs; OBA, opioid-based anesthesia; OFA, opioid-free anesthesia; PBM, patient blood management; PGE2, prostaglandin 2; p.p., percentage points; PRBC, packed red blood cells; RCT, randomized controlled trial; SIRS, systemic inflammatory response syndrome; SLUG, SNAIL-related zinc-finger transcription factor; SSR, surgical stress response; TAMs, tumor-associated macrophages; TANs, tumor-associated neutrophils; TGF, tumor growth factor; Th1/2, T helper 1/2; TME, tumor microenvironment; TNF, tumor necrosis factor; TLR, toll-like receptor; TRIM, transfusion-related immunomodulation; VEGF, vascular endothelial growth factor.
